# Differential Effects of Mindfulness-Based Intervention Programs at Work on Psychological Wellbeing and Work Engagement

**DOI:** 10.3389/fpsyg.2021.715146

**Published:** 2021-09-27

**Authors:** Cristián Coo Calcagni, Marisa Salanova, Susana Llorens, Miguel Bellosta-Batalla, David Martínez-Rubio, Rosa Martínez Borrás

**Affiliations:** ^1^Department of Developmental, Educational & Social Psychology and Methodology, Jaume I University, Castellón de La Plana, Spain; ^2^Department of Personality, Evaluation and Psychological Treatment, University of Valencia, Valencia, Spain; ^3^Departamento de Psicología, Universidad Europea de Valencia, Valencia, Spain; ^4^Koesencia Consulting, Santiago de Compostela, A Coruña, Spain

**Keywords:** mindfulness at work, psychological wellbeing, work engagement, performance, stress, intervention

## Abstract

Two different mindfulness-based interventions were deployed in a sample of white-collar workers to explore the differential effects on different facets of mindfulness, dimensions of psychological wellbeing, work engagement, performance, and stress of a participant. A total of 28 participants completed one of the different programs, and their results were compared between groups and against 27 participants randomly allocated to a waiting list control group. Results suggest both mindfulness intervention programs were successful at increasing the levels of psychological wellbeing, work engagement, and performance of the participants, as well as decreasing their levels of stress. Significant differences were found between the two programs in all outcome variables. Results suggest that brief and customized mindfulness interventions at work are as successful as lengthier programs.

## Introduction

Mindfulness is defined as an inherent ability of the human mind to pay attention to present moment experiences adopting an attitude characterized by curiosity, openness, and acceptance (Bishop et al., 2004). Different theoretical models of mindfulness propose it is composed of different elements or “facets” that can be developed as skills through systematic training deployed in the format of mindfulness-based interventions (MBIs) (Carmody and Baer, 2008; Creswell, 2017; Lindsay et al., 2018a,b; Lindsay and Creswell, 2019; Sansó et al., 2019). Workplace-delivered MBI programs are increasingly showing to be an effective strategy to help employees manage stress and improve their mental health (Eby et al., 2016; Bartlett et al., 2019). The majority of published studies on MBIs at work focuses on decreasing stress and mental health-related outcomes such as anxiety, psychological distress, and burnout (Lomas et al., 2017), and the systematization of the available data initially supports this claim (Heckenberg et al., 2018). As well, mindfulness seems to have an impact both on physiological and psychological pathways that explain these effects (Chiesa et al., 2011; Lao et al., 2016). Overall, it seems to be a promising strategy to address a wide array of problems that arise from the characteristics of many of the jobs and workplaces of today (Good et al., 2016).

However, the available knowledge on MBIs at work is limited in at least three crucial aspects. First, most of the MBI evaluation studies in a work-related setting focus mainly on healthcare workers (Bartlett et al., 2019). Due to the nature of their work, they experience high levels of job demands and increasing levels of psychosocial risks that lead to conditions such as depressive symptoms, compassion fatigue, and burnout (Pisljar et al., 2011; Gleichgerrcht and Decety, 2014; Alexandrova-Karamanova et al., 2016; Parola et al., 2017). This scenario makes healthcare workers ideal candidates to test the alleged effects of MBIs; particularly more so in this moment in time when healthcare systems worldwide are under greater levels of pressure than ever. Unfortunately, this bias has produced a lack of studies focusing on different working populations, such as white-collar workers. The term “white-collar” worker was coined during the 1930s in the US reflecting the usual attire (white shirt and tie) of individuals in professional occupations that required a higher level of education than traditional manual labor. Traditionally, white-collar workers consider job occupations dedicated to performing managerial, professional, or administrative work. Different occupations included in this broad category are executive management, management consulting, human resources, information technology, research, and technology among many others (Van Horn and Schaffner, 2003). White-collar workers comprise the majority of the workforce in services-driven economies (EUROSTAT, 2017; U.S Bureau of Labor Statistics, 2019). They are also exposed to significant levels of job demands and psychosocial risks due to the preeminently mental rather than physical effort associated with the characteristics of their work (Bridger and Brasher, 2011; Fila et al., 2017). Thus, they are also good candidates to benefit from MBIs at work. Services-based organizations may benefit from it in the form of increased performance and productivity, and decreased levels of stress-derived health complications in their workforce.

A second limitation has to do with the relatively small number of studies inquiring about outcomes related to mental health that go beyond the simple reduction of negative aspects of human experience such as stress, depression, and anxiety. Mental health is not only related to the absence of disease but also the presence of wellbeing (WHO, 2005). When it comes to MBIs, it is necessary to adopt a more holistic perspective of mental health the includes “non-clinical” approaches such as psychological wellbeing and work engagement (Ivtzan et al., 2016). Psychological wellbeing is a multidimensional construct that englobes different aspects of life, such as meaning, relations, and personal growth (Ryff and Singer, 2008). These aspects are represented by specific domains or “dimensions” that are distinct from one another but taken together reflect the different elements that make up a “good life” (van Dierendonck et al., 2007). Along a similar line, work engagement poses a work-specific approach to psychological wellbeing that is characterized by high levels of energy and willingness to invest effort in the work of the individual, experiencing a sense of enthusiasm, pride, and challenge, and being fully concentrated and happily engrossed in the work of the individual (Schaufeli et al., 2002). Although a distinct construct on its own (Schaufeli and Salanova, 2011), work engagement could be considered a domain-specific measure of psychological wellbeing. As well, it reflects the eudaimonic component of psychological wellbeing in the sense that it is related to sustained effort, motivation, and optimal functioning (Straume and Vittersø, 2014). Psychological wellbeing and work engagement are not only relevant in terms of health but also regarded as critical aspects to attain a better performance both at the individual (Lyubomirsky et al., 2005; Zelenski et al., 2008) and organizational levels (Taris and Schreurs, 2009; Salanova et al., 2012; Salanova and Llorens, 2016).

A third limitation of work-related MBIs literature has to do with the scarcity of measurements of performance and productivity. Mindfulness has been positively associated with different improvements in cognitive ability and emotional regulation as possible pathways to improve performance (Chiesa et al., 2011; Holzel et al., 2011). As well as with specific work-related concepts such as sunken cost bias (Hafenbrack et al., 2014). Preliminary evidence suggests there might be a positive effect of mindfulness on performance but more research on this relation is needed in order to clarify the benefits of MBIs in regard to this element (Good et al., 2016; Kersemaekers et al., 2018).

Taken together, the three distinct limitations mentioned above make a strong case for the development and evaluation of MBIs deployed at work that focus on different samples beyond healthcare workers, that include measurements of well-being both with a broad perspective and contextual specificity, and that incorporate to the very least some measure of performance.

Finally, it is necessary to stress the fact that there is a wide variety of different MBIs available ranging from fully standardized programs (Kabat-Zinn, 2013) to full-on customizations (Wolever et al., 2012). This poses an important dilemma when choosing what type of MBI protocols to deploy, and striking a balance between commitment to established guidelines and customization to improve adherence and success becomes a challenge on its own. Callings for refinement in MBI intervention research point out the value of utilizing standardized intervention protocols when possible while at the same advocating for the development of specific MBI protocols adapted to specific workplace characteristics and needs of the worker (Lomas et al., 2017). In this sense, there is a significant gap related to the evaluation of differential effects between established MBI programs compared to customized MBI versions developed for specific contexts and populations.

In light of the established gaps existing in regard to the MBIs at work literature, we propose the present study. The aim is to test the differential effects of two types of MBIs at work. More specifically to compare a customized, and brief work-specific MBI program with a longer duration MBI program based on the MBCT (Segal et al., 2001) and self-compassion (Neff, 2003; Barnard and Curry, 2011) in a white-collar worker population, looking at the potential differences on the effects of participants’ levels of mindfulness, psychological wellbeing, work engagement, stress, and performance.

Considering the existing literature on MBIs at work, and their positive impact on levels of mindfulness, different measures of wellbeing (i.e., subjective psychological wellbeing, work engagement, and job satisfaction among others; Lomas et al., 2017), performance (Coo and Salanova, 2018), and diminishing stress (Bartlett et al., 2019), we propose the following hypotheses.

### Hypotheses

*H*1: Both MBI programs (i.e., MSCBI and MPSM) will increase the levels of different facets of mindfulness (i.e., acting with awareness) of participants in comparison with participants in the control group.

*H*2: Both MBI programs (i.e., MSCBI and MPSM) will increase the levels of different dimensions psychological well-being (i.e., environmental mastery) and work engagement (i.e., vigor) of participants in comparison with participants in the control group.

*H*3: The MBCT-based program (MSCBI) will be more effective at increasing the levels of different faces of mindfulness of participants and diminishing their levels of stress.

*H*4: The MBI work-specific program (MPSM) will be more effective at increasing the levels of different dimensions of work engagement (i.e., vigor) and performance (i.e., in-role performance) of participants.

## Materials and Methods

### Participants and Procedure

Workers from two different organizations (Organization A and Organization B) in the industrial production area were invited to participate in distinct MBI programs as workplace initiatives to manage stress and enhance wellbeing. More specifically, workers from management and back-office areas were the target group invited to participate fitting the “white-collar” category described above. All of them performed either administrative, operations or, managerial desk-bound duties, and 30% held management positions with teams under their supervision. Recruited participants were screened for pre-existing conditions such as ongoing psychiatric treatments, depression, and anxiety, in which case they were advised to consult with their therapists whether participation in the activity was advised. Afterward, participants were distributed between either an intervention or waiting-list control group following a randomization procedure. Participants allocated to the waiting-list control group took part in the different intervention programs once the first intervention group and data collection process were finished. Participation was voluntary and no compensation was offered upon the enlistment or completion of the program.

Both participating organizations were based in Spain, were large in size with more than 250 workers each, yielding 50€ million or more annually in net revenue. Organization A was a company dedicated to manufacturing and distribution of construction materials and supplies on a large scale. Organization B was a company dedicated to engineering and manufacturing supplies for the automobile industry.

For Organization A, participants answered a paper-based questionnaire prior to the beginning of the intervention program and 1 week after the last training session. For Organization B, participants were asked to answer an online questionnaire distributed *via* e-mail previous to the beginning of the intervention programs, and 1 week after the last session of the program. The questionnaire included an informed consent form complying with the latest data management regulations, and the study was sanctioned and approved by the first author’s host university ethics committee.

Organization A offered a 6-week MBI based on the MBCT (Segal et al., 2001) standardized intervention including a component of self-compassion (Neff, 2003) labeled “Mindfulness and Self-Compassion Intervention” (MSCBI); Organization B offered a brief 3-week MBI custom program integrating MBCT (Segal et al., 2001; Kuyken et al., 2010) and ACT (Hayes et al., 2006) labeled “Mindfulness and Positive Stress Management” (MPSM). The content and rationale of both MBI programs can be found in Tables 1, 2.

**TABLE 1:** MPSM intervention program specific session content and structure.

**Session No.**	**Length in hours**	**Rationale**	**Structure**	**Homework**
1	4	• What is stress? Personal experiences, physical and emotional correlates. • Physiology of the stress response and its relation to human evolution. Fight, Flight or Freeze. • What is mindfulness? Brief body scan exercise, sharing personal experiences. • Definition and established benefits of mindfulness practice, and self-directed neuroplasticity. • Mindfulness and stress management through de-centering and re-appraisal of stressful situations.	• Class orientation (Welcome, Format, Intentions). • Ground rules • Introductions. • Experiences of Stress and brief presentation. • Body scan. • Benefits of mindfulness and mechanisms of action. • Sitting meditation with focus on breath • Re-appraisal exercise.	• Body scan and/or sitting meditation. • Mindfulness of routine activity. • Practice log.
2	4	• Mindfulness and character strengths. Mindfulness as a pathway to cultivating our best-possible self. • Understanding and discovering our signature strengths as well as those we would like to develop. • Identifying strengths in action, exploring new ways of practicing them, and imagining new pathways to cultivate new strengths. • Using strengths to overcome obstacles and difficult situations.	• Brief body scan check-in. • Home practice review. • Mindfulness and character strengths introduction. • Discover, identify and. practice personal strengths. • Explore and establish new behaviors to practice strengths	• Body scan and/or mindfulness of routine activity. • Mindful character strengths practice. • Practice log.
3	4	• Identifying areas of balance/unbalance in our work life. • Identifying patterns of recurring thoughts/behaviors that lead to stress and difficulty • Balancing character strengths with mindfulness practice for optimal use. • Developing specific action plans to address and transform our patterns into professional and personal growth opportunities. • Exploring our best possible self into the future as a guideline to follow in our professional and personal growth. • Choosing intentional and committed actions to cultivate our inner and outer balance.	• Brief body scan check-in. • Homework review • Balance/Unbalance in our working life • Balancing character strengths • Action plan development • Best possible self • Final thoughts	• Body scan and/or mindfulness of routine activity. • Mindful character strengths practice. • Best possible self in balance. • Practice log.

**TABLE 2:** MSCBI intervention program specific session content and structure.

**Session No.**	**Length in hours**	**Rationale**	**Structure**	**Homework**
1	2	• Reflecting on the social context and our daily habits. • How does our mind work? Attentional default network and the automatisms present in our mind. • Identifying the contents of the mind: thoughts, emotions and feeling. Decentering • What is mindfulness? • Formal and informal practice	• Class orientation (Welcome, Format, Intentions). • Ground rules • First mediation practice. Observing our inner experience and motivation. Why are we here? • Introductions. • Practice. What does our mind do when it is doing nothing? • What is mindfulness? Basic concepts introduction • Raisin mindful eating meditation • Collective reflection and conclusions. Instructions to keep practicing during the week.	• Brief pauses during the day (1–3 min). What are you doing? How do you feel? What are you thinking? • Mindfulness of breathing and awareness of inner experience (7–10 min). • Mindful eating • Practice Log
2	2	• Reflection on the main obstacles for practice • Understanding how to calm our mind. Focused attention on our body. Our breath as our ally. • Differentiating the Self as a subject and the self as an object. • Mechanisms of action and benefits of practice.	• Body scan (10 min) • Group reflection on the main obstacles while trying to practice at home. • Monitoring hand movements. • Group reflection on the different perspectives of the self (subject vs. object). • Mechanism of action. Benefits from a neurophysiological, mental and behavioral perspective. From reaction to choice. • Collective reflection and conclusions. Instructions to keep practicing during the week.	• Brief pauses during the day (1–3 min). • Body scan, calm and hand monitoring mediations. • Informal practice of daily activities • Daily gratefulness and practice log
3	2	• How to train a stable mind? Attention regulation. • Learning to stabilize or mind through mindfulness of breathing. • Identifying the right attitude in mindfulness practice. • Developing other forms of being present in our daily life. • Identifying the link between thoughts and emotions.	• Mindfulness of breathing. • Review of homework. • Attention stability and breathing as a regulator. • What kind of attitude to maintain during practice? • Mindful movement and walking • Mindfulness of breathing focusing on the belly. • Observation and experimentation. Mindful eating black chocolate. Where I put my attention, I create my reality. • Collective reflection and conclusions. Instructions to keep practicing during the week.	• Brief pauses during the day (1–3 min). • Mindfulness of breathing. Observing thoughts, and mindful movement (15 min). • Informal practice of daily activities • Mindfulness of social media and tv consumption • Gratefulness Letter • Practice Log
4	4	• Being present through our senses. Broadening our perspective. • Training our mind for clarity. • Knowing our relation with our thoughts. • Exploring acceptance and differentiating between primary and secondary pain. • Interpersonal mindfulness, mindful listening and talking.	• Mindfulness of the 5 senses including thoughts. • Review of homework. • Presentation on mental clarity. • Mindfulness of nose focused breathing. • Presentation on acceptance and primary and secondary pain. • Mental experiment Yes/No repeat. • Mindful listening and talking in couples. • Collective reflection and conclusions. Instructions to keep practicing during the week.	• Mindfulness of the 5 senses including thoughts. Acceptance and Openness. • Informal practice of daily activities. Mindful listening. • Mindfulness of difficulties and resistance. Practicing letting go. • Practice Log
5	4	• Basic skills for wellbeing. • Identifying emotional balance systems: Alert, achievement and connection. • Developing empathy. • Understanding compassion and self-compassion. • Developing gratitude.	• Mindfulness at the end of the day. • Review of homework. Main obstacles and difficulties. • Emotional regulation system by Gilbert. • Self-Compassion model by Neff. • Presentation and reflection on compassion and self-compassion, impermanence of relations, and video. • Mindfulness of self-care • Collective reflection and conclusions. Instructions to keep practicing during the week.	• Mindfulness at end of the day and self-care. • Kindness toward oneself and others. • Support videos. • Random acts of kindness. • Practice Log.
6	4	• Compassion and adherence to practice. • Last reflection and clearing doubts about compassion. • Distinguishing between different kinds of relations. Broadening circles. • Acquiring guidelines to sustain our practice.	• Mindfulness of gratitude. • Review of homework. • Presentation and reflection on compassion. • Kindness and compassion mediation in couples. • Group reflection on key learning points. • Guidelines to keep practicing independently in our daily lives • Collective reflection and conclusions.	• Kindness and compassion, as well as any other of the exercises practices during the course. • Autonomous weekly practice group. • Maintaining what we learned.

A total of 22 participants were allocated in the MBI program offered by Organization A, from now on labeled as MBSR Group, 13 of them completed the intervention program and the pre-post evaluation. They were 45.5 (SD = 7.25) years on average and 41.4% were women. A total of 20 participants were allocated in the MBI program offered by Organization B, from now on labeled as MPSM Group. Of the initial group, 15 participants completed the program and pre-post evaluation. They averaged 41 years of age (SD = 6.92) and 52% were women. Finally, 18 participants from Organization A and 15 participants from Organization B were allocated to the waiting list control group, for a total of 33 participants in the control group. They were 38.5 (SD = 10.72) years old on average and 51% were women. Cronbach’s *α* and correlations for all variables at pre- and post-intervention times are shown in Tables 3, 4.

**TABLE 3:** Cronbach’s α and correlations for all sub scales at pre intervention time.

		**α**	**1**	**2**	**3**	**4**	**5**	**6**	**7**	**8**	**9**	**10**	**11**	**12**	**13**	**14**	**15**
Mindfulness (FFMQ)	Acting with awareness	0.88	–														
	Describe	0.79	0.018	–													
	Non-Judgment	0.68	0.052	–0.158	–												
	Non-Reactivity	0.75	0.121	0.154	0.135	–											
Subjective Psychological Wellbeing (SPWB)	Self-Acceptance	0.73	0.189	0.172	0.339**	0.163	–										
	Positive Relations	0.75	0.284*	0.224	0.182	0.178	0.477**	–									
	Autonomy	0.77	0.134	0.223	0.411**	0.241	0.489**	0.484**	–								
	Environmental Mastery	0.67	0.323**	0.276*	0.139	0.272*	0.662**	0.526**	0.548**	–							
	Purpose in Life	0.84	0.204	0.287*	0.153	0.119	0.642**	0.508**	0.541**	0.699**	–						
	Personal Growth	0.71	0.148	0.124	0.021	0.118	0.334**	0.333**	0.313*	0.416**	0.407**	–					
Work Engagement	Vigor	0.78	0.126	–0.141	–0.134	0.007	0.139	0.049	–0.020	0.252*	0.379**	0.162	–				
	Absorption	0.88	0.104	–0.195	0.075	–0.011	0.058	0.025	0.039	0.077	0.155	0.275*	0.526**	–			
	Dedication	0.83	0.115	0.026	–0.122	–0.012	0.200	0.131	0.005	0.287*	0.332**	0.196	0.801**	0.495**	–		
Performance	In-role Performance	0.76	0.012	–0.028	–0.091	0.067	0.013	–0.002	–0.039	–0.164	–0.023	0.094	0.040	0.327**	–0.013	–	
	Extra-role Performance	0.74	–0.118	0.015	–0.015	–0.117	0.253*	–0.003	0.109	0.177	0.306*	0.122	0.419**	0.367**	0.422**	0.449**	–
Stress		0.73	–0.108	–0.171	0.139	0.235	0.111	–0.075	–0.035	–0.173	–0.109	0.060	–0.002	–0.091	0.049	0.076	–0.002

*p < 0.05; **p < 0.01.

**TABLE 4:** Cronbach’s α and correlations for all sub scales at post-intervention time.

		**α**	**1**	**2**	**3**	**4**	**5**	**6**	**7**	**8**	**9**	**10**	**11**	**12**	**13**	**14**	**15**
Mindfulness (FFMQ)	Acting with-awareness	0.78	–														
	Describe	0.80	0.219	–													
	Non-Judgment	0.83	0.622**	0.108	–												
	Non-Reactivity	0.72	0.225	0.196	0.285*	–											
Subjective Psychological Wellbeing (SPWB)	Self-Acceptance	0.85	0.134	0.443**	0.043	0.332*	–										
	Positive Relations	0.72	0.232	0.362*	0.157	0.313*	0.351*	–									
	Autonomy	0.72	0.430**	0.264	0.213	0.154	0.144	0.498**	–								
	Environmental Mastery	0.78	0.437**	0.301*	0.286*	0.139	0.322*	0.660**	0.627**	–							
	Purpose in Life	0.84	0.004	0.369**	–0.006	0.220	0.733**	0.417**	0.154	0.398**	–						
	Personal Growth	0.75	0.275	0.286*	0.141	0.111	0.477**	0.685**	0.533**	0.750**	0.510**	–					
Work Engagement	Vigor	0.83	0.271	0.042	0.151	0.022	–0.068	0.230	0.204	0.384**	0.074	0.191	–				
	Absorption	0.92	0.455**	0.157	0.399**	0.183	0.249	0.259	0.411**	0.337*	0.245	0.219	0.634**	–			
	Dedication	0.83	0.128	0.153	0.151	0.116	0.169	0.248	0.198	0.302*	0.334*	0.260	0.724**	0.631**	–		
Performance	In-role Performance	0.87	0.160	0.088	0.216	–0.073	0.034	0.415**	0.194	0.357*	0.074	0.228	0.370**	0.440**	0.358*	–	
	Extra-role Performance	0.83	0.232	0.264	0.131	0.143	0.277	0.441**	0.184	0.294*	0.270	0.274	0.510**	0.597**	0.636**	0.654**	–
Stress		0.79	−0.312*	0.186	−0.290*	–0.084	0.188	–0.112	–0.266	–0.027	0.317	–0.002	0.033	–0.013	0.101	–0.027	0.014

*p < 0.05; **p < 0.01.

### Program Descriptions

Mindfulness and positive stress management or MPSM is a customized intervention program combining core elements of the traditional mindfulness teachings with specific tools from the field of positive psychology (i.e., character strengths) combined inside the framework or stress-management from a proactive perspective. This program aimed to develop specific and tailored action plans oriented to managing recurring sources of stress from an adaptative perspective. On the other side, mindfulness and self-compassion intervention or MSCBI is a more traditional program following a standardized format oriented specifically to developing mindfulness skills and tools combined with compassion with the explicit goal of enhancing wellbeing through mental training.

Both programs share the basic core of mindfulness teachings and skills but differ in the specific goals, framework, and strategies to deploy and transfer the skills and tools to everyday life.

Mindfulness and positive stress management focuses on developing a set of core skills and provides an established step-by-step guide to deploy said skills as core elements with a clear and committed goal. MSCBI offers a wider and more exploratory approach to mindfulness built on self-experimentation through different meditation techniques in a wide variety of everyday scenarios.

As well, MPSM explicitly introduces the concept of character strengths and values in action (Peterson and Seligman, 2004) as key elements to develop congruent and committed goals and action plans. On another side, MSCBI introduces such concepts in a more implicit manner when exploring the topics of wellbeing and compassion.

Finally, MPSM is delivered in a brief and condensed three-session length format that seeks to explicitly tackle the main sources of recurring stress in everyday work-related situations. MSCBI follows the traditional 8-week format focusing on building a regular and sustained mindfulness practice that is not directed at any particular type of event but to life in general.

### Measures

*Mindfulness* was measured using the Spanish validation of the five facet mindfulness questionnaire (FFMQ; Baer et al., 2006; Cebolla et al., 2012; Coo Calcagni and Salanova Soria, 2016). It is a 20-item short version scale that assesses five different dimensions of mindfulness understanding it as a higher order factor. The five dimensions comprise, namely, Observe (OBS), Describe (DES), Act with Awareness (AW), Non-Reactivity to own thoughts (NR), and Non-Judgment to own experience (NJ). Participants indicate the frequency of 20 behaviors on a 7-point Likert scale (0 = *almost never*, 6 = *almost always*). Items include “I’m good at finding words to describe my feelings” and “I’m easily distracted.” Half of the items are reverse scored. Following Baer et al. (2008) we decided to exclude the Observe subscale to facilitate the detection of training-related changes in mindfulness. The scale presented good internal reliability.

*Psychological Wellbeing* was measured using the short version Spanish adaptation of the Psychological Wellbeing Scale (SPWB; Díaz et al., 2006; Ryff and Singer, 2008). The 29-item scale assesses six distinct domains of wellbeing (Self-acceptance [SE], Positive relations [PR], Autonomy [AT], Environmental mastery [EM], Purpose in life [PL], and Personal growth [PG]). Participants rate their levels of agreement/disagreement regarding different statements using a six-point Likert scale (1 = *totally disagree*; 6 = *totally agree*). Sample items include “I feel like many of the people I know have gotten more out of life than I have” [SE], “Most people see me as loving and affectionate” [PR], “I have confidence in my opinions even if they are *contrary to the general consensus*” [AT], “I am good at juggling my time so that I can fit everything in that needs to get done” [EM], “I enjoy making plans for the future and working to make them a reality” [PL], and “I have the sense that I have developed a lot as a person over time” [PG]. The scale presented good internal reliability.

*Work Engagement* was measured using the Spanish version of the Utrecht Work Engagement Scale in its 9-item version (UWES9; Schaufeli and Bakker, 2003; Schaufeli et al., 2006). The scale is composed of three dimensions: (I) Vigor, (II) Dedication, and (III) Absorption. Participants indicate the frequency of specific feelings and behaviors on a 6-point Likert scale (1 = *almost never*, 6 = *almost always*) including “At my job, I feel strong and vigorous” and “I’m enthusiastic about my job.” The scale presented good internal reliability.

*Performance* was measured using the six-item scale from Goodman and Svyantek (1999) that assesses in-role and extra-role performance using a 7 point Likert type scale (0 = *almost never*, 6 = *almost always*). The items include, “I achieve my work-related objectives” and “I go beyond my official responsibilities to help my teammates.” The scale showed acceptable internal reliability.

*Stress* was measured using the Spanish validation of the Perceived Stress Scale in its 10-item version (Trujillo and González-Cabrera, 2007; Cohen et al., 2014). Participants respond to the frequency of specific statements about thoughts and feelings during the previous month on a 5-point Likert scale (0 = never, 5 = very often). Sample items include, “During the last month How frequently have you felt nervous or stressed?”. The scale acceptable internal reliability.

### Data Analysis

First, a one-way ANOVA test was conducted to establish sufficient baseline similarity for all variables between the three groups (MPSM, MSCBI, and Control). Non-significant results for this would allow for further comparison of the intervention effects including post-intervention measurements for all groups.

Second, to analyze the effects of the different MBI protocols, we conducted a multivariate ANOVA (MANOVA) with a 3 × 2 (Group × Time) design with three distinct group conditions (MPSM, MSCBI, and Control) as our between-subjects variables and two-time points of measurement (pre- and post-intervention) including all outcome variables. To a finer-grained description of the differential effects we introduced each one of the outcome variables per sub-scales [e.g., For Mindfulness, we used the sub-scales of Describe (DES), Act with Awareness (AW), Non-Reactivity to own thoughts (NR), and Non-Judgment to own experience (NJ)].

With the MANOVA analysis, we seek to observe the differences in the mean scores of each one of the outcome variables across the different groups. The effect represented by time will reflect if the MBI protocols were effective from a general perspective, the group effect will point out if there exist any differences between groups at the general mean level, and the interaction term of group × time will establish if there are differences related to the type of intervention participants underwent and its effects.

Effect sizes were calculated using eta-squared (*η*^2^) and Cohen’s *d* with specific cut-off points established at 0.02, 0.13, and 0.26, for small, medium, and big effects, respectively (Cohen, 1992).

## Results

As a first step, demographics and outcome variables were compared across groups at the baseline level (pre-intervention). There were no significant differences across groups with regard to gender distribution *s*^2^(3) = 1.723, *p* = 0.632. As well there were no significant differences in age groups distribution between groups *χ*^2^(9) = 9.058, *p* = 0.432. Finally, there were no significant differences between the different groups for all the outcome subscales of the variable, for specific results see Table 5.

**TABLE 5:** Pre-intervention one-way ANOVA test with group as comparison factor.

**Scales**	**Dimensions**	**df_effect_**	**df_error_**	**F**	***P***
Mindfulness (FFMQ)	Describe	2	61	0.15	0.857
	Act with Awareness	2	61	2.02	0.140
	Non-Judgment	2	61	1.18	0.316
	Non-Reactivity	2	61	0.54	0.586
Psychological wellbeing (SPWB)	Self-acceptance	2	61	0.18	0.839
	Positive relations	2	61	0.12	0.890
	Autonomy	2	61	0.84	0.436
	Environmental mastery	2	61	0.49	0.618
	Purpose in life	2	61	0.66	0.523
	Personal growth	2	61	0.98	0.380
Engagement (UWES)	Dedication	2	61	0.06	0.946
	Vigor	2	61	0.40	0.961
	Absorption	2	61	0.53	0.590
Performance	In role Performance	2	61	0.20	0.821
	Extra Role Performance	2	61	1.00	0.905
Stress (PSS)		2	61	1.70	0.192

Second, with the MANOVA we observed the effects for the time, group, and the interaction term of time × group. Results indicate a significant effect for a time along with big effect size, Pillai’s trace = 0.683, *F*(16,31) = 4.174, *p* < 0.001, *η*^2^ = 0.684, suggesting significant changes in all groups across time. For group, significant effect a big effect size was found, Pillai’s trace = 1.206, *F*(32,64) = 3.035, *p* < 0.001, *η*^2^ = 0.603, indicating the three are significant differences across all groups. Last, there was a significant effect for the interaction term time × group with big effect size, Pillai’s trace = 1.287, *F*(32,64) = 3.509, *p* < 0.001, *η*^2^ = 0.643, indicating the changes across time are related to the type of intervention participants took part off.

**FIGURE 1:**
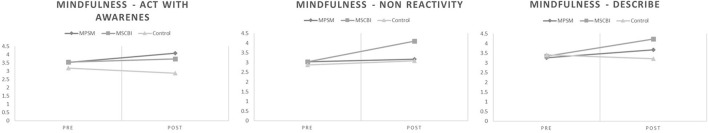
Means estimated for the MBSR group, MPSM group, and control group on pre-intervention and post-intervention time points, for mindfulness dimensions with statistically significant interaction effect.

Third, we analyzed the follow-up ANOVAs for each one of the outcome variables specific sub-scales representing their dimensions to establish detailed differences between groups. First, we analyzed the sub-scales corresponding to mindfulness. Looking at the results of the time × group interaction, results suggest that significant differences between groups across time could be observed for the sub-scales of Describe [*F*(2,46) = 4.342, *p* = 0.019, *η*^2^ = 0.159], Act with Awareness [*F*(2,46) = 4.342, *p* = 0.024, *η*^2^ = 0.149] and Non-reactivity [*F*(2,46) = 5.032, *p* = 0.011, *η*^2^ = 0.180], all with large effect sizes. No significant differences of mean scores between groups cross-time were detected for the sub-scale of Non-judgment [*F*(2,46) = 1.819, *p* = 0.174, *η*^2^ = 0.073]. Results are of follow-up ANOVAs are shown in Table 6. Close inspection of mean scores suggests that the MSCBI group was more effective at increasing the Describe and Non-reactivity dimensions of mindfulness while the MPSM group was more effective at increasing Acting with Awareness. Mean scores and standard deviations are shown in Table 7. These effects become more evident when looking at the graphical representation of the interaction term presented in Figure 1. In light of these results, we deem Hypothesis 1 supported and established partial support for Hypothesis 3.

**TABLE 6:** Follow-up ANOVA test for the effects of time, group and their interaction on outcome variables.

		**Time**					**Group**					Time*Group				
**Scales**	**Dimensions**	**df_effect_**	**df_error_**	**F**	***P***	***η2***	**df_effect_**	**df_error_**	**F**	***P***	***η2***	**df_effect_**	**df_error_**	**F**	***P***	***η2***
Mindfulness (FFMQ)	Describe	1	46	3.423	0.071	0.069	2	46	1.413	0.254	0.058	2	46	4.342	0.019	0.159
	Act with Awareness	1	46	1.703	0.198	0.036	2	46	12.858	<0.001	0.359	2	46	4.035	0.024	0.149
	Non-Judgment	1	46	0.650	0.424	0.014	2	46	4.740	0.013	0.171	2	46	1.819	0.174	0.073
	Non-Reactivity	1	46	21.057	<0.001	0.314	2	46	10.515	<0.001	0.314	2	46	5.032	0.011	0.180
Psychological Wellbeing (SPWB)	Self-acceptance	1	46	12.126	0.001	0.209	2	46	0.778	0.465	0.033	2	46	1.358	0.267	0.056
	Positive relations	1	46	1.357	0.243	0.021	2	46	3.004	0.059	0.116	2	46	5.815	0.006	0.202
	Autonomy	1	46	4.245	0.045	0.084	2	46	9.162	<0.001	0.295	2	46	3.261	0.047	0.124
	Environmental mastery	1	46	2.012	0.163	0.042	2	46	6.598	0.003	0.223	2	46	5.375	0.008	0.189
	Purpose in life	1	46	0.276	0.602	0.042	2	46	0.404	0.670	0.017	2	46	0.248	0.782	0.011
	Personal growth	1	46	2.341	0.080	0.034	2	46	1.925	0.157	0.077	2	46	3.472	0.094	0.055
Engagement (UWES)	Dedication	1	46	3.670	0.62	0.074	2	46	0.106	0.899	0.005	2	46	2.644	0.082	0.103
	Vigor	1	46	2.376	0.130	0.049	2	46	0.568	0.571	0.024	2	46	15.189	<0.001	0.398
	Absorption	1	46	26.371	<0.001	0.364	2	46	2.183	0.124	0.087	2	46	11.000	<0.001	0.324
Performance	In role Performance	1	46	0.749	0.391	0.016	2	46	0.741	0.482	0.032	2	46	5.211	0.009	0.185
	Extra Role Performance	1	46	6.628	0.013	0.126	2	46	1.320	0.277	0.054	2	46	3.336	0.044	0.127
Stress (PSS)		1	46	3.271	0.077	0.066	2	46	0.178	0.837	0.008	2	46	4.667	0.014	0.169
																

**TABLE 7:** Pre – Post-intervention and control groups scores– mean (SD).

		**Intervention group [MPSM]**		**Intervention group [MSCBI]**		**Control group**	
**Scales**	**Dimensions**	**Pre**	**Post**	**Pre**	**Post**	**Pre**	**Post**
Mindfulness (FFMQ)	Describe	3.27(1.16)	3.67(1.08)	3.35(0.80)	4.23(0.59)	3.42(0.49)	3.22(1.29)
	Act with Awareness	3.52(0.48)	4.08(0.83)	3.54(1.05)	3.74(0.66)	3.17(0.42)	2.88(0.50)
	Non-Judgment	3.53(0.46)	3.64(0.56)	3.68(0.99)	3.89(0.91)	3.38(0.41)	3.11(0.62)
	Non-Reactivity	3.03(0.48)	3.17(0.41)	3.03(0.78)	4.10(0.63)	2.88(0.36)	3.08(0.71)
Psychological wellbeing (SPWB)	Self-acceptance	4.45(0.88)	4.83(0.76)	4.60(0.90)	5.06(0.52)	4.55(0.55)	4.63(0.85)
	Positive relations	4.13(0.78)	4.57(0.79)	4.15(1.11)	4.43(0.82)	4.26(0.81)	3.48(0.93)
	Autonomy	3.98(0.91)	4.63(0.50)	3.65(1.08)	4.07(0.63)	3.61(0.78)	3.31(0.86)
	Environmental mastery	4.26(1.10)	4.96(0.39)	4.21(0.79)	4.15(0.49)	4.04(0.59)	3.85(0.67)
	Purpose in life	4.49(1.00)	4.52(1.02)	4.77(0.76)	4.78(0.85)	4.55(0.75)	4.69(0.79)
	Personal growth	5.45(4.09)	5.43(0.44)	4.56(0.95)	4.73(1.00)	4.60(0.67)	4.57(0.63)
Engagement (UWES)	Dedication	4.31(0.82)	4.64(0.70)	4.39(0.90)	4.58(0.99)	4.40(0.81)	4.56(0.88)
	Vigor	4.35(0.79)	4.97(0.54)	4.39(0.60)	4.43(0.99)	4.42(0.73)	4.32(0.68)
	Absorption	4.11(0.74)	4.80(0.69)	3.87(0.59)	4.64(1.07)	4.00(0.71)	3.90(0.75)
Performance	In role Performance	5.08(0.58)	5.17(0.46)	5.03(0.50)	5.25(0.57)	5.12(0.49)	4.89(0.45)
	Extra Role Performance	4.70(1.00)	5.16(0.63)	4.58(0.83)	5.07(0.92)	4.59(0.82)	4.57(0.84)
Stress (PSS)		2.72(0.53)	2.33(0.33)	2.57(0.37)	2.30(0.43)	2.47(0.40)	2.65(0.33)
							

**FIGURE 2:**
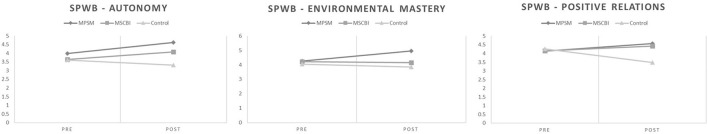
Means estimated for the MBSR group, MPSM group, and control group on pre-intervention and post-intervention time points, for spwb dimensions with statistically significant interaction effect.

For the sub-scales of subjective psychological wellbeing, the results suggest that significant differences between groups across time could be observed for the sub-scales of Positive Relations [*F*(2,46) = 5.815, *p* = 0.006, *η*^2^ = 0.202], Autonomy [*F*(2,46) = 3.261, *p* = 0.047, *η^2^* = 0.124] and Environmental Mastery [*F*(2,46) = 5.375, *p* = 0.008, *η^2^* = 0.189], once again with large effect sizes for all the variables. On the contrary, no significant effects were observed for the sub-scales of Self-Acceptance [*F*(2,46) = 1.358, *p* = 0.267, *η*^2^ = 0.056], Purpose in Life [*F*(2,46) = 0.248, *p* = 0.782, *η*^2^ = 0.011] and Personal Growth [*F*(2,46) = 3.472, *p* = 0.094, *η^2^* = 0.055]. When looking at the different groups means scores, the MPSM group was more effective at increasing the al three dimensions that showed significant differences. A graphical representation of the results is shown in Figure 2. In summary, these results support Hypothesis 2.

**FIGURE 3:**
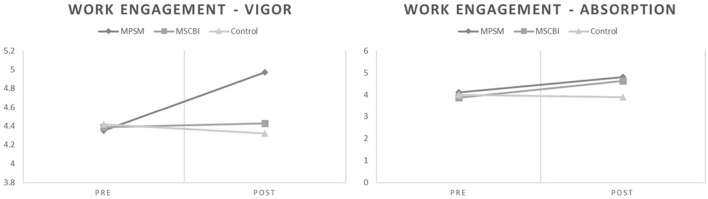
Means estimated for the MBSR group, MPSM group, and control group on pre-intervention and post-intervention time points, for work engagement dimensions with statistically significant interaction effect.

Concerning the sub-scales of work engagement, significant differences between groups across time could be observed for the sub-scales of Vigor [*F*(2,46) = 15.189, *p* ≤ 0.001, *η*^2^ = 0.011] and Absorption [*F*(2,46) = 11.000, *p* ≤ 0.001, *η*^2^ = 0.324], but not for Dedication [*F*(2,46) = 2.644, *p* = 0.082, *η*^2^ = 0.103] with large sized effects for all variables. When observing the mean scores of different groups, the MPSM group was more successful at increasing both dimensions of work engagement. Furthermore, this effect is graphically represented in Figure 3. These results provide partial support to Hypothesis 4.

**FIGURE 4:**
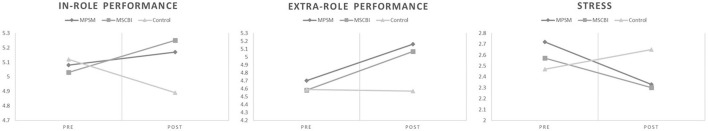
Means estimated for the MBSR group, MPSM group, and control group on pre-intervention and post-intervention time points, for performance dimensions and perceived stress with statistically significant interaction effect.

For the sub-scales of performance, both In Role Performance [*F*(2,46) = 5.211, *p* = 009, *η*^2^ = 0.185] and Extra-Role Performance [*F*(2,46) = 3.336, *p* = 044, *η*^2^ = 0.127] exhibited significant differences between groups across time with large size effects. Closer inspection of mean scores suggests that the MSCBI group was more effective at increasing In-Role Performance, while on the other hand, the MPSM group was more effective at increasing Extra-Role Performance. A graphical representation of this effect is presented in Figure 4. In line with the previous paragraph, these results provide full support for Hypothesis 4.

Finally, significant differences between groups across time were observed for Stress [*F*(2,46) = 4.667, *p* = 014, *η*^2^ = 0.169]. Mean scores suggest that the MSCBI group was slightly more effective at reducing stress than the MPSM group. Graphical representation of this effect is also presented in Figure 4. In addition, with the results related to the differences between groups on the different dimensions of Mindfulness, these results provide full support to Hypothesis 3.

## Discussion

This study aimed to observe the effects of two different types of MBIs (a lengthier MSCBI program, and a custom work-specific MPSM program) on the levels of mindfulness, psychological wellbeing, work engagement, performance, and stress in a sample of white-collar workers who belonged to two different organizations.

To begin, we seek to establish basic levels of efficacy for both MBI intervention programs when comparing them to a waiting-list control group. Overall, the results suggest that both MBI programs were effective at increasing the levels of mindfulness, psychological wellbeing, work engagement, and performance, as well as diminishing the effects of stress when compared to the control group. The results are in line with previous research and build the case for MBI at work as a successful strategy to increase employee health and wellbeing from a broad perspective that goes beyond the pure amelioration of negative aspects of experience.

Looking into the differential effects of each one of the programs, the MSCBI program was significantly better at increasing the mindfulness facets of description and non-reactivity toward inner experience. This suggests that both the length and depth of the program could be important factors to develop specific mindfulness-related skills. Traditional MBI programs may be better suited for this particular task due to those specific factors.

Nonetheless, the custom MPSM program proved to be slightly better at increasing the acting with awareness mindfulness facet. In the case of this particular difference, we hypothesize it might be related to the use of psychoeducation methods and exercises in tandem with mediation practices that could enhance the sense of awareness present experience. An example of this is the use of character strengths emphasizing self-observation of specific values and behaviors, as well as establishing concrete action plans in the exploration of new ways to practice signature strengths as a mindfulness curiosity exercise.

Concerning the different dimensions of psychological wellbeing, the MPSM program turned out to be slightly better than the MSCBI program at increasing the facets of autonomy, environmental mastery, and positive relations. In this particular case, we believe the narrative focus from ACT (Hayes et al., 2006), which builds around the development of intentional and values-committed actions, adds a significant explicit difference that accounts for this difference when it comes to increased levels of autonomy and environmental mastery. Deliberate focus on developing new behaviors related to personal values and positive characteristics of the self may have a more significant impact on the sense of autonomy since it is related to evaluating oneself according to personal standards (Ryff and Singer, 2008). MSCBI is also related to specific actions to detect maladaptive patterns of behavior related to stress. Still, these do not necessarily come explicitly in the form of approach goals or developing new behaviors. Along the same line, the capacity of feeling a sense of control over complex and changing scenarios reflected by environmental mastery is present in the MPSM rationale. The elaboration of specific action plans to cope and re-appraise difficult situations utilizing the inner resources of the participants is a perfect example of this idea. About the changes in positive relations, the differentiation of effects is not as clear between both programs since the differences are marginal. Both programs are deployed in a group setting that invites participants to share personal experiences and insights, incorporating vicarious learning experiences as an important factor.

About the differences in work engagement, our predictions were clearly supported by the results. The differential effects were particularly more explicit on the dimension of vigor. We believe this effect to be related to the incorporation of character strengths in tandem with mindfulness. Individuals who act upon their personal strengths tend to be more energized and engaged (Peláez et al., 2019). For the dimension of absorption, the differences are barely noticeable, and even though the scores for the participants from the MPSM group are a little higher in the post-intervention measurement time, the participants of the MSCBI group saw a more substantial increase from pre to post measurements. Thus, we believe the differences are not so relevant in this particular aspect.

With regard to performance, the results showed that the MSCBI program was slightly better at increasing in-role performance. More prolonged exposure to systematic meditation practices can be a significant factor when accounting for this difference. Executive processing and attentional capacities that change with meditation practices are dose-dependent (Lao et al., 2016), therefore a larger dose may have a significantly larger effect in the specific processes that may support individual in-role performance. On the other hand, the MBSP program was better at increasing extra-role performance, which revolves around behaviors that go above and beyond established goals and responsibilities. Again, we believe this is linked to the incorporation of character strengths to mindfulness practice since individuals that have the possibility to practice and enact their values in work-related scenarios tend to go beyond the norm in terms of effort and engagement with their work and companions (Peláez et al., 2019).

Next, the results regarding the decrease in levels of stress are in line with the existing literature pointing to the benefits of MBIs as effective strategies to help workers manage stress (Khoury et al., 2015; Bartlett et al., 2019). In this line, it is no surprise that the MSCBI program was more effective at diminishing the stress of the participants, considering that the rationale and focus of the program are built around this particular goal. However, it is important to note that shorter programs can be successful as well. They should be treated as the initial steps in the stress management process and should be sustained in time utilizing complementary strategies such as workplace-based wellbeing promotion programs that underline the importance of sustained practice in time to reap the benefits of mindfulness.

Finally, this study presents significant contributions to the study of MBIs in the workplace setting. First, it expands the effectiveness of MBIs to the population of office or white-collar workers proving that not only healthcare workers can benefit from mindfulness and related skills in their daily activities at work. Second, we support the claim for the positive effects of MBIs beyond the mitigation of negative aspects of human experience and broaden the scope toward the inclusion of wellbeing-related constructs such as work engagement and psychological wellbeing. This proposal is aligned with the calling for bridges between contemplative traditions and psychology articulated toward the pursuit of our highest potential or best possible self (Cebolla et al., 2017; Coo and Salanova, 2018). Third, we provide evidence in favor of mindfulness changing the individuals’ perception of their own capacity and performance. This claim is not only rooted in subjective experience changes in relation to work capacity of the workers but also in the changes that occur in terms of stress management, executive processing, and cognitive flexibility improvements, and their neurophysiological correlates (Holzel et al., 2011).

Last but not least, our study supports the use of customized MBIs adapted to the work context. Even though there is great value in the use of standardized programs, adaptations of the basic building blocks of MBI to the experience of the participants are key when designing interventions for success. Underlying this notion is the fact that not all intervention designs will work the same for different groups of people, and thus is valuable work to establish legitimate and effective customized approaches that take into account what works from whom under what circumstances (Nielsen and Miraglia, 2016).

### Limitations

Besides the contributions our study seeks to offer, there are also a significant number of limitations. First, the use of solely self-report measures is one of the recurring weaknesses of intervention studies in general. In our case, we could not access objective measures of performance, nor implement behavioral measures of mindfulness due to constraints imposed by the organizations we worked with. However, in an effort to provide an argument in favor of the validity of our data and following the recommendations of Podsakoff et al., 2012) to address common-method bias, single latent factor tests were performed for both pre and post measurements and in both cases the amount of variance explained by the unrotated single factors solution was less than 20% indicating the distinctiveness of each measure.

Another critical limitation has to do with the small size of the samples. Intervention studies require a great deal of time and resources from the researchers and the participating organizations, and expanding sample sizes toward the inclusion of larger numbers of participants is an endeavor that requires an equally large amount of time, resources, and effort. That being said, smaller sample sizes of well-described and contextualized scenarios are still valuable and pose a Contribution To The Field.

Finally, the lack of long-term follow-up measurements hinders our ability to test the longevity of the effects of the different intervention protocols. Discriminating confounding and contextual effects with the passage of time in different workplaces makes it difficult to support the validity of long-term measurements. However, the development and inclusion of objective measures of performance, and biobehavioral aspects of wellbeing can be a potential solution for this predicament.

### Future Research and Final Remarks

As for suggestions toward future research, we believe there is great value in the design and implementation of intervention studies that incorporate different blocks of content and skills to be developed that allows for testing in a scaled fashion between different groups to dismantle the effects of different components (e.g., Lindsay et al., 2018b). Approaches like this could shed light on the possible synergies between different components, clearly identifying the core aspects of MBIs and also looking for potentially unwanted effects.

As well, the inclusion of cost-effect evaluations is the logical next step to develop solid arguments that go beyond the psychological benefits of implementing MBIs at work. Including financial evidence in favor of MBI as occupational health interventions with a positive return on investment will make them more readily available both in the private and public sectors.

Finally, incorporating objective measures of performance and biobehavioral aspects of wellbeing can further legitimate the positive effects derived from MBIs at work, providing solid ground for the actual benefits going beyond experimental and laboratory settings. Along the same line, planning for long term follow-up measures in tandem with structural measures to improve adherence to practice and effect sustainability is a relevant area still to be explored as is the incorporation of a multilevel perspective to expand the conception of mindfulness beyond the individual perspective into teams and organizations.

## Data Availability Statement

The raw data supporting the conclusions of this article will be made available by the authors, without undue reservation.

## Ethics Statement

The studies involving human participants were reviewed and approved by the Research Ethics Committee of Universitat Jaume I of Castellón, Spain. The patients/participants provided their written informed consent to participate in this study.

## Author Contributions

CC contributed with the concept, data collection, analysis, writing, and editing of the manuscript. MS, SL, and DM-R contributed to the concept and review of the manuscript. MB-B contributed with the data collection and treatment. RM contributed with the intervention program design and description. All authors contributed to the article and approved the submitted version.

## Conflict of Interest

RM was employed by company Koesencia Consulting. The remaining authors declare that the research was conducted in the absence of any commercial or financial relationships that could be construed as a potential conflict of interest.

## Publisher’s Note

All claims expressed in this article are solely those of the authors and do not necessarily represent those of their affiliated organizations, or those of the publisher, the editors and the reviewers. Any product that may be evaluated in this article, or claim that may be made by its manufacturer, is not guaranteed or endorsed by the publisher.
